# Comparative Analysis of *Listeria monocytogenes* Plasmids and Expression Levels of Plasmid-Encoded Genes during Growth under Salt and Acid Stress Conditions

**DOI:** 10.3390/toxins11070426

**Published:** 2019-07-20

**Authors:** Patricia Hingston, Thomas Brenner, Lisbeth Truelstrup Hansen, Siyun Wang

**Affiliations:** 1 Department of Food, Nutrition and Health, University of British Columbia, Vancouver, BC V6T 1Z4, Canada; 2 National Food Institute, Technical University of Denmark, 2800 Kongens Lyngby, Denmark

**Keywords:** plasmid gene expression, plasmid characterization, salt tolerance, acid tolerance, food safety, *Listeria monocytogenes*

## Abstract

*Listeria monocytogenes* strains are known to harbour plasmids that confer resistance to sanitizers, heavy metals, and antibiotics; however, very little research has been conducted into how plasmids may influence *L. monocytogenes*’ ability to tolerate food-related stresses. To investigate this, a library (*n* = 93) of *L. monocytogenes* plasmid sequences were compared. Plasmid sequences were divided into two groups (G1 and G2) based on a *repA* phylogeny. Twenty-six unique plasmid types were observed, with 13 belonging to each of the two *repA*-based groups. G1 plasmids were significantly (*p* < 0.05) smaller than G2 plasmids but contained a larger diversity of genes. The most prevalent G1 plasmid (57,083 bp) was observed in 26 strains from both Switzerland and Canada and a variety of serotypes. Quantitative PCR (qPCR) revealed a >2-fold induction of plasmid-contained genes encoding an NADH peroxidase, cadmium ATPase, multicopper oxidase, and a ClpL chaperone protein during growth under salt (6% NaCl) and acid conditions (pH 5) and ProW, an osmolyte transporter, under salt stress conditions. No differences in salt and acid tolerance were observed between plasmid-cured and wildtype strains. This work highlights the abundance of specific plasmid types among food-related *L. monocytogenes* strains, the unique characteristics of G1 and G2 plasmids, and the possible contributions of plasmids to *L. monocytogenes* tolerance to food-related stresses.

## 1. Introduction

*Listeria monocytogenes* is a ubiquitous, facultatively anaerobic, foodborne pathogen that is capable of growing at temperatures as low as −0.4 °C [1], at remarkably low pH [2], and in high osmolarity environments [3], making it particularly dangerous as a foodborne pathogen; especially with regards to refrigerated, ready-to-eat foods. Furthermore, foodborne listeriosis infections are exceptionally dangerous to high-risk individuals, causing an unprecedented average mortality rate of ~30% [4].

Like many bacterial species, *L. monocytogenes* strains are known to harbour plasmids with frequencies reaching as high as 79% [5,6,7,8,9,10]. To date, plasmids acquired by *L. monocytogenes* have been shown to contain genes that confer resistance to sanitizers [11,12,13], heavy metals [7,12,13], and to a range of common antibiotics, including chloramphenicol, clindamycin, erythromycin, streptomycin and tetracycline [14,15]. Additionally, genes associated with oxidative, osmotic, and heat stress have also been observed in *L. monocytogenes* plasmids [16,17,18].

Interestingly, higher rates of plasmid harbourage have been reported among food and environmental isolates compared to clinical isolates [7,19]. Furthermore, plasmid harbourage rates have been shown to be higher amongst reoccurring strains of *L. monocytogenes* (75%) as opposed to sporadic strains (35%) isolated from food or food-processing facilities [20], suggesting that genes found in *L. monocytogenes* plasmids may be beneficial for the survival of this pathogen in such environments. For example, *L. monocytogenes* isolates containing the plasmid-encoded *bcrABC* genes quadruple the MIC of benzalkonium chloride, a common ammonium-based food industry disinfectant, compared to plasmid cured strains [11]. Frequent application of such disinfectants in the food industry may create a selective pressure favouring the uptake of *brcABC* containing plasmids from other *L. monocytogenes* strains and thus lead to a higher prevalence among food-related isolates. Many heavy metal-resistance genes for cadmium, copper, lead, zinc, mercury and arsenic have also been described in *L. monocytogenes* plasmids. While mostly plasmid-mediated, cadmium resistance has been demonstrated to date [7,21,22] carriage of chromosomally encoded *cadA3* and *cadA4* have been reported in the sequenced EGDe strain and a few other strains and in strains harbouring the Listeria Genomic Island 2 (LGI2), respectively [23,24,25]. Such genes may provide benefits for survival in natural environments as heavy metals are often found in both soil and water [26,27,28].

Plasmid harbourage and subsequent replication poses a metabolic burden on cells and has been shown to lead to decreased growth rates relative to plasmid-cured strains [29]. However, plasmid-harbourage likely provides cells with a growth advantage when exposed to selective conditions. In a previous study [30], we found that *L. monocytogenes* strains possessing a plasmid had significantly faster growth rates in acidified media (pH 5) compared to plasmid-free strains, prompting the hypothesis that genes found in *L. monocytogenes* plasmids contribute to acid tolerance. Furthermore, when plasmid-positive strains were categorized into two groups based on a *repA* phylogeny, the replicon gene used to indicate plasmid presence as suggested by Kuenne et al. [16], strains harbouring the larger group 2 plasmids had significantly faster growth rates in 6% NaCl than strains with the smaller group 1 plasmids. Given the importance of being able to control the growth of *L. monocytogenes* in foods, these findings warrant a deeper investigation into the roles that plasmids may play in the growth and survival of *L. monocytogenes* in foods. 

The objectives of this study were to determine the contribution of genes found in *L. monocytogenes* plasmids to the bacterium’s ability to survive food-related stresses (acid and salt) and to also assess the genetic commonalities and differences between *repA* sequence-based plasmid groups.

## 2. Results and Discussion

### 2.1. L. monocytogenes Plasmid Types and Characteristics 

Among the 93 plasmid sequences, 26 unique plasmid types were detected with 13 belonging to group 1 (G1) and 13 belonging to group 2 (G2) based on their *repA* profile (Table 1; Table 2). It was observed that many of the strains from our Canadian and Swiss collaborative collection, harboured the same plasmid types with pLMG1-7 in particular, being identified in 26 strains from both Canada and Switzerland and covering four different serotypes and seven different clonal complexes (CC). A high degree of similarity among some *Listeria* plasmid nucleotide sequences has been reported in other studies [16,31]. However, in these cases, it was certain DNA regions such as harborage of the Tn5422 transposon, that were highly similar rather than whole plasmid sequences. On the other hand, Schmitz-Esser et al. [32] reported that nine whole-genome sequences available for sequence type (ST) 121 *L. monocytogenes* strains, harboured near identical 62–63 kbp plasmids. Indeed, the only sequence type (ST) 121 strain in our collection contained a plasmid with 100% identity to that found in strain Lm6179 analyzed in Schmitz-Esser et al. [32]. Schmitz-Esser et al. [32] highlighted that near-identical plasmids were found in ST121 strains isolated from different sources, countries, and years, suggesting a strong selective pressure maintains the presence of ST121 plasmids. In the present study, identical plasmids were not only observed among strains from the same location or sero-, sequence or clonal complex (CC) type, but also across these categories (Table 1 and Table 2), further emphasizing the astounding level of plasmid sequence conservation and spread among *L. monocytogenes* strains. 

In our previous study [30], G1 plasmids were found to be significantly (*p* < 0.0005) smaller (26–88 kbp) than G2 plasmids (55–107 kbp). Accordingly, in the present study, G1 plasmids were found to contain 29–113 predicted genes whereas G2 plasmids encoded 63–120 genes. The G + C contents of the two plasmid groups also differed slightly with G1 plasmids containing 34.4–36.9% GC, and G2 plasmids having slightly higher GC levels of 36.6–37.7%. Despite the slight differences, all GC levels align with those previously reported for *L. monocytogenes* plasmids [17,32] and genomes (37–38% GC) [33], as well as the genomes of other firmicutes such as *Lactobacillus*, *Staphylococcus*, and *Clostridium* species [34]. A Blastn search of all plasmid contigs only revealed similarities to other *Listeria* spp. plasmids.

One interesting observation that we noted was that G2 plasmid sequences consisted of 1–3 contigs, whereas G1 plasmid sequences consisted of ≤ 7 contigs. Furthermore, plasmid contigs were found to be identical across all strains harbouring the same plasmid types. The number of contigs resulting from software-assembled genomic sequences largely depends on the frequency of repeat elements such as prophages, transposases, and IS elements as these sections are often difficult to assemble [35]. Accordingly, G1 plasmids contained more mobile genetic elements than G2 plasmids (Table 2 and Table 3), despite G2 plasmids being larger in size. 

**Figure 1:**
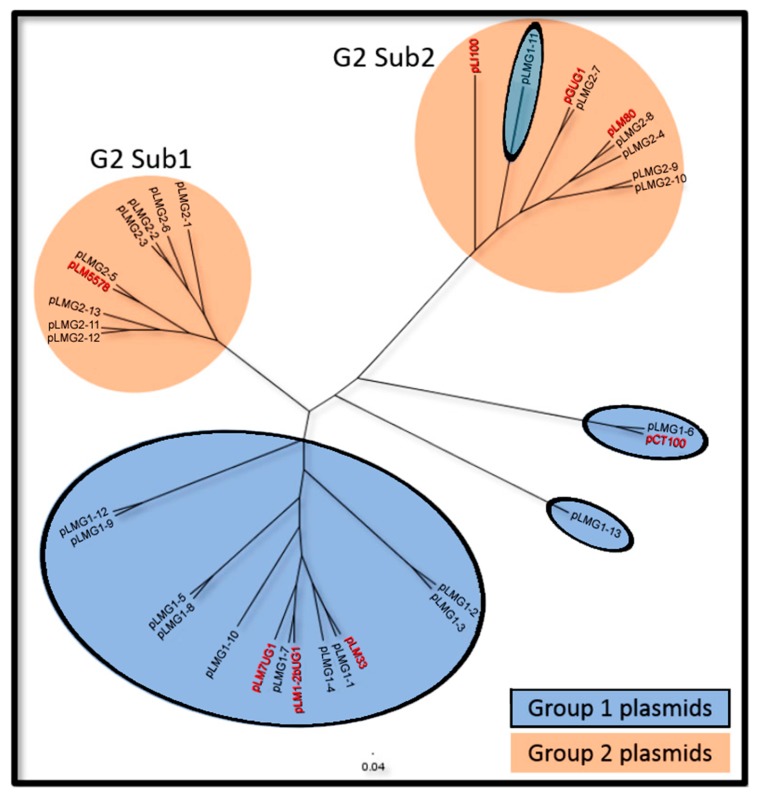
A neighbour-joining phylogenic tree showing the genetic groupings of the *Listeria* group 1 (G1) and group 2 (G2) plasmids. Plasmid types highlighted in red were used in Kuenne et al. [16].

A complete sequence phylogeny of all plasmid types from the present study, and eight reference plasmids from other studies revealed three distinct groupings (Figure 1). The first group consisted of 10 of our G1 plasmids and three G1 plasmids from Kuenne et al. [16], whereas the second and third groups represented two discrete subgroups of G2 plasmids (Sub1 and Sub2, Figure 1). Of the four remaining G1 plasmids, pLmG1-6 and pCT100 were closely related to only each other, pLMG1-13 formed its own unique cluster, and pLMG1-11 grouped with Sub2 G2 plasmids. The genetic similarities and differences between the different plasmid clusters will be further discussed.

### 2.2. Genetic Elements Shared by Group 1 and Group 2 Plasmids

Three predicted genes were found in all plasmid types: *repA*, responsible for plasmid replication; *repB,* responsible for plasmid partitioning; and an excinuclease ABC subunit A which is involved in DNA repair. These same three genes were also found in all 14 *Listeria* sp. plasmids investigated in Kuenne et al. [16] and on most plasmids, all three genes were located in the same region. In G1 plasmids the three genes were all separated by one hypothetical protein, whereas in G2 plasmids there was no hypothetical protein between *repA* and *repB*. On three plasmids (pLMG1-11, pLMG2-11, and pLMG2-13), the excinuclease was located far apart from the *repA* and *repB* region. It should also be noted that G1 and G2 plasmids shared a large number of identical genes encoding hypothetical proteins of unknown function, emphasizing how much we still have to learn regarding *L. monocytogenes* plasmids.

**Figure 2:**
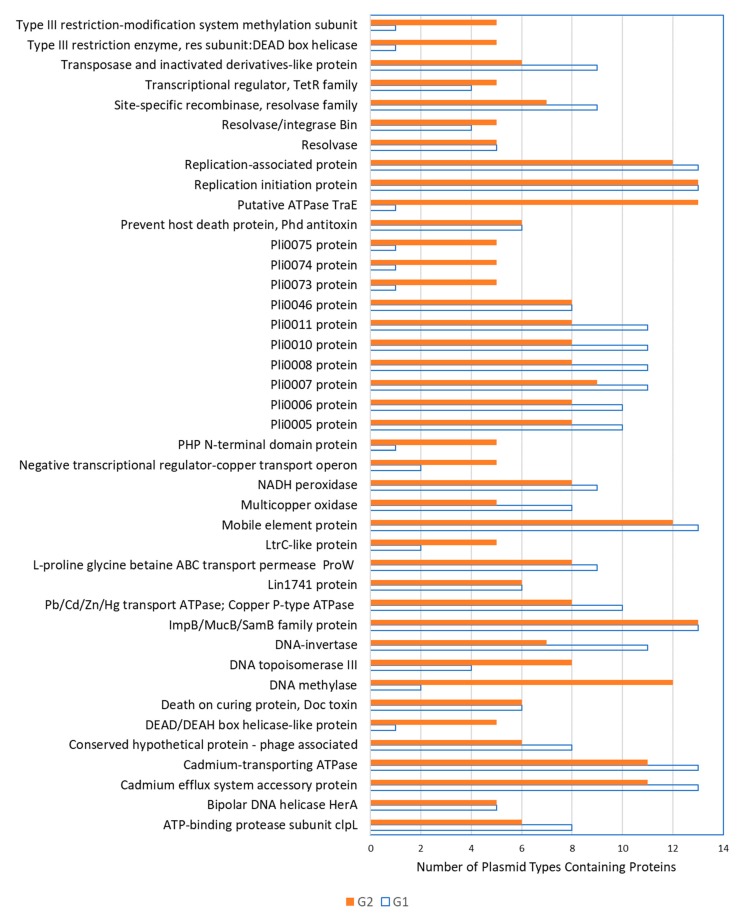
The predicted proteins observed in both group 1 and group 2 plasmids appearing on ≥ 5 plasmid types (*n =* 13 plasmid types for both G1 and G2).

**Figure 3:**
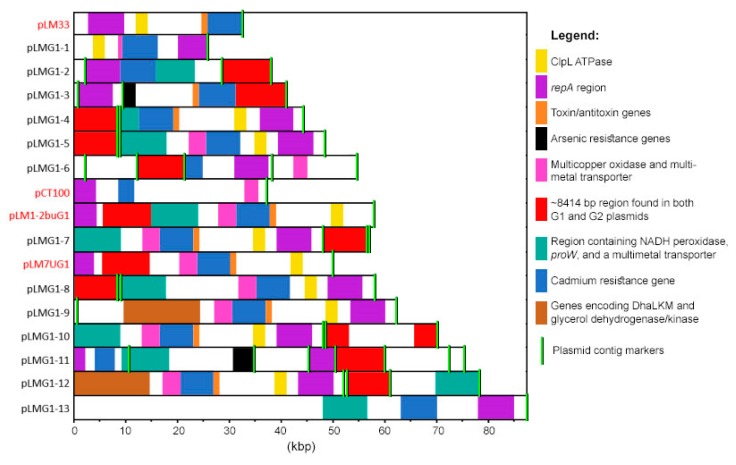
The alignment of *L. monocytogenes* G1 plasmid sequences including 13 G1 concatenated plasmid sequences from this study and four additional sequences from Kuenne et al. [16], highlighted in red font. Regions filled in with the same colour share a high degree of genetic similarity. It should be noted that Mauve was not able to identify all homologies due to algorithmic limitations.

**Figure 4:**
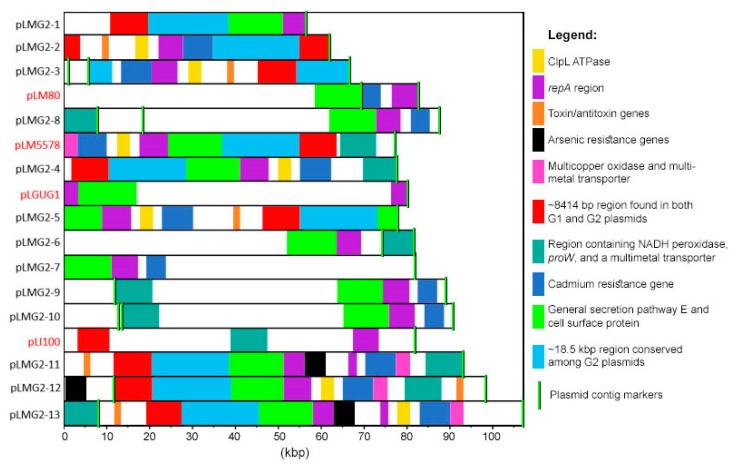
The alignment of *L. monocytogenes* G2 plasmid sequences including 13 G2 concatenated plasmid sequences from this study and four additional sequences used in Kuenne et al. [16], highlighted in red font. Regions filled in with the same colour share a high degree of genetic similarity. It should be noted that Mauve was not able to identify all homologies due to algorithmic limitations.

Other genetic features were also highly prevalent in both G1 and G2 plasmids (Figure 2), including versions of a cadmium-transporting ATPase (*cadA*) and of a cadmium efflux system accessory protein (*cadC*). These predicted genes were found in all 13 G1 plasmid types and in 11 out of 13 G2 plasmid types. Similarly, in Kuenne et al. [16], all but one of their plasmids (pLGUG1) contained two cadmium resistance genes. In the present study, two different versions of the genes existed. Eleven G1 and seven G2 plasmids (Table S2) contained both cadmium resistance genes (*cadA1* and *cadC1*) on the Tn5422 transposon which additionally encodes two transposases [36]. This transposon is present on numerous *Listeria* sp. plasmid sequences available in the NCBI database. An additional two G1 and four G2 plasmid types (Table S2) contained *cadA2* and *cadC2.* These genes were originally detected on the pLM80 plasmid [11] and, in fact, all four G2 plasmid types containing *cadA2* contained the entire pLM80 plasmid whereas this was untrue for the two G1 plasmid types. A number of plasmids from both the G1 and G2 groups also contained a multi-metal transporter and a multicopper oxidase adjacent to the cadmium resistance genes (Figure 2, Figure 3 and Figure 4).

Although cadmium resistance genes also exist on *Listeria* sp. chromosomes, they are more commonly found in plasmids [37]. Interestingly, plasmid-encoded resistance genes have been reported to result in greater tolerance to cadmium compared to the chromosomally encoded *cadA4* gene found within LGI2 [24]. Five (A144, Lm212, Lm50, Lm116, Lm236) of 166 *L. monocytogenes* strains in our collection were found to possess chromosomally encoded *cadA4*, with Lm116 and Lm236 both additionally harbouring the pLMG2-10 plasmid which contains *cadA2.* The strains harbouring pLMG2-10 were serotype 1/2a, clonal complex (CC) 204 while the remaining three strains were serotype 4b, CC2 in which LGI2 is prevalent [25]. Four additional strains contained a variant of *cadA4* that shares 85% of the nucleotide identity and 88% of coverage. In agreement with previous literature [38], three (Lm69, Lm77, Lm88) of these strains were serotype 4b, CC1 while one (Lm220) represented a novel serotype 4b sequence type. Lastly, all strains containing the pLMG2-7 plasmid carried the Tn5422 cadmium resistance (*cadA1*) transposon in their chromosome, suggesting that the transposon likely migrated from the plasmid to the chromosome in these strains.

Other predicted genes highly prevalent among both G1 and G2 plasmids included those encoding an NADH peroxidase, an L-proline glycine betaine ABC transport system permease protein (ProW), and a lead, cadmium, zinc and mercury transporting ATPase (Figure 2). These genes have also been reported on other *L. monocytogenes* plasmids [16,17]. All three genes were located close together on the plasmids with the NADH peroxidase and *proW* existing adjacent to one another, followed by three hypothetical proteins and then the multi-metal transporting ATPase. Mobile transposable elements flanked at either end of this entire region highlighting the transferability of these genes.

Ten G1 plasmid types (pLMG1-2 to 8 and pLMG1-10 to 12) and seven G2 plasmid types (pLMG2-2 to 3, pLMG2-5 to 6, and pLMG2-11 to 13) contained a ~8414 bp region that existed either as part of the *repA* containing contig, or as an individual separate contig (Figure 3; Figure 4). On all G2 plasmids, the region was located on the *repA* contig and contained 10 complete open-reading-frames (ORFs) and two additional ORFs on either end of the region that was incomplete in some plasmid types. Eight of the 10 complete ORFs encoded hypothetical proteins and the remaining two encoded a DNA binding protein and a cell filamentation protein (Fic). When complete versions of the two bordering ORFs existed, they encoded a predicted DNA methylase and a hypothetical protein.

The arsenic resistance cassette previously identified in pLI100 [16], was found in two G1 and three G2 plasmid types in this study (Table S2, Figure 3; Figure 4). Interestingly, all strains harbouring pLMG1-1 to 2, pLMG1-4 to 5, and pLMG1-8 plasmid types, as well as three strains harbouring pLMG1-7 plasmid types, contained the arsenic resistance cassette on a chromosomal contig as opposed to on a plasmid. Four additional plasmid-free strains also contained this cassette. While *L. monocytogenes* chromosomes have been previously shown to harbour this arsenic resistance cassette [37], its presence has not yet been associated with strains harbouring specific plasmid types. The fact that all strains harbouring specific G1 plasmid types contained this cassette suggests that the cassette was likely at some point part of these plasmids and migrated into the chromosome.

Another notable observation from the plasmid comparison analysis was the presence of the PemIK toxin/antitoxin stable maintenance system in the six G1 and six G2 plasmids (Figure 3; Figure 4). In the G1 plasmids, these genes were located adjacent to the cadmium resistance cassette. This toxin-antitoxin system has been described for the *Lactobacillus salivarius* UCC118 plasmid pSF118–20 [39] and was also found to be present on many of the *Listeria* sp. plasmids analyzed by Kuenne et al. [16]. Several studies have documented the roles of plasmid-encoded toxin-antitoxin systems in controlling the loss or maintenance of plasmid-harbourage in the absence of selective environmental pressures [40,41,42,43,44,45] which is likely responsible for the prevalence of these systems across many plasmid types. Other genes found in both plasmid groups included those encoding a clpL ATPase, DNA helicase (HerA), DNA topoisomerase III, a number of transcription regulators, and a putative ATPase (TraE) (Table 4).

Consistent with previous literature regarding *L. monocytogenes* plasmids [10,16,19,20], no antibiotic resistance genes were identified on our plasmid sequences. While *L. monocytogenes* plasmids have been found to contain genes that facilitate resistance to chloramphenicol, erythromycin, streptomycin, and tetracycline [14,15], such incidences remain rare.

### 2.3. Genetic Elements Associated with Group 1 Plasmids

A total of 43 predicted genes were uniquely observed in G1 plasmids (Table 1). The most prevalent G1-specific genes encoded a CRISPR-associated protein and two hypothetical proteins from *L. monocytogenes* EGE-e which were found in five plasmid types belonging to the main G1 cluster. Otherwise, G1-specific genes were only found in one or two plasmid types. A second toxin-antitoxin system containing a zeta toxin and epsilon antitoxin gene was found in two G1 plasmid types (Table 1). This system has also been reported on streptococcal and enterococcal plasmids [46,47]. A third plasmid type additionally contained a RelE/StbE replicon stabilization toxin and a RelB/StbD replicon stabilization antitoxin system that has been reported on *Escherichia coli*, *Bartonella ancashensis*, and *Klebsiella pneumoniae* plasmids among others [48,49,50].

Other notable predicted genes specific to G1 plasmids included those encoding alcohol dehydrogenase, glycerol dehydrogenase and glycerol kinase with collective roles in glycerol utilization; a copper-transporting ATPase; a CrcB protein which aids in removing fluoride from cells; a mercury resistance cassette; a MATE family multidrug resistance protein; a phosphate regulatory protein (PhoB); three protein subunits of a phosphoenolpyruvate-dependent dihydroxyacetone (Dha) kinase (DhaKLM), and an ATP-dependent Dha kinase (Table 1). On two plasmid types (pLmG1-9 and 12), the alcohol and glycerol dehydrogenases and glycerol kinase occurred adjacent to the genes encoding DhaKLM, two ATP-dependent Dha kinases, and a TetR transcription regulator with mobile genetic elements flanking either end of the entire region (Figure 3). TetR has been shown to activate the transcription of *dhaKLM*, and glycerol dehydrogenase and kinase are used to convert glycerol into Dha, one of the simplest carbohydrates, making it an important precursor for the synthesis of organic compounds in bacteria [51,52]. Strains harbouring plasmids with this region may have improved access to carbon which may be used for the production of energy or cellular constituents such as membrane phospholipids, which could potentially enhance the ability of a strain to tolerate certain stresses.

Overall, many G1 plasmids contained similar sets of predicted genes with the exception of three G1 plasmid types that were not part of the main G1 cluster shown in Figure 1. pLMG1-6 formed its own cluster with pCT100, an *L. monocytogenes* G1 plasmid from Kuenne et al. [16]. These two plasmids share a unique region that encodes an additional multi-metal transporter, a copper-transporting ATPase, a copper operon negative transcriptional regulator, and a MATE family multidrug resistance protein (Figure 3). Another plasmid, pLMG1-13, did not group with any other plasmid types analyzed in this study and more than half of the predicted genes were unique to this plasmid type (Table 3).

### 2.4. Genetic Elements Associated with Group 2 Plasmids

On average, G2 plasmids contained more genes than G1 plasmids (90 vs. 67); however, there was less diversity among G2 plasmid genes, presumably due to the lower number of mobile genetic elements. Unique to G2 plasmids was the presence of predicted genes encoding virulenc-associated proteins, as well as a cell surface protein, a general secretion pathway E protein, and a membrane-bound protease which were all found in ≥8 G2 plasmid types (Table 4).

All eight G2 plasmids containing the previously described 8414 bp region found in both G1 and G2 plasmids, had an adjacent region of approximately 18.5 kbp that contained predicted genes encoding a lipoprotein, ATP TraE, DNA topoisomerase III, a membrane-bound protease, and a type IV secretory pathway (Figure 4). All of these plasmids belonged to G2 sub1 in Figure 1, and with the exception of pLMG2-3, the 18.5 kbp region lied directly adjacent to another G2 specific region of ~12 kbp that encoded a general secretion pathway E protein and a cell surface protein (Figure 4). This 12 kbp region was prevalent among almost all G2 plasmid types with pLMG2-2 and pLMG2-3 being exceptions. However, the general secretion pathway E protein was retained in these plasmids despite the absence of the remaining parts of the 12 kbp region.

Within the G2 plasmid subgroups (Sub1 and Sub2), many plasmid types shared a high degree of genetic similarity. For example, pLMG2-2 differed from pLMG2-1 by only the presence of a ClpL ATPase and a cadmium resistance transposon (Tn5422), and pLMG2-3 differed from pLMG2-2 by only the presence of a mobile genetic element encoding the multidrug efflux pumps EbrA and EbrB, along with a TetR transcription regulator. EbrAB belongs to the SMR family of multidrug efflux pumps and in *E. coli* and *Bacillus subtilis*, they have been shown to be responsible for resistance to ethidium, acriflavine, pyronine Y, safranin O and tetraphenylphosphonium (TPP) chloride [53].

Within G2 Sub1 plasmids, pLMG2-13, the largest plasmid type in our sequence collection, differed from pLMG2-11 and pLMG2-12 by the presence of a region encoding a predicted DNA topoisomerase III, mannose-6-phosphate isomerase, YbbM seven transmembrane helix protein, ferroxidase, apolipoprotein diacyclglyceryl transferase, and a copper chaperone and copper ATPase. This region lies directly downstream of the multicopper oxidase and multi-metal transporter genes in pLMG2-13 (Figure 4). This same region was also found in pLMG1-8. pLMG2-11 and pLMG2-12 differed only by the presence of *clpB* encoding a stress-induced chaperone protein, and the region containing NADH peroxidase and *proW*. Similarly, pLMG2-10 differed from pLMG2-6 by only the presence of an arsenic resistance cassette.

Plasmids from G2 Sub2 were unique in that they all contained a ~43 kbp region containing a large number of predicted genes including those encoding VirB4 and VirD4 secretion system proteins and an invasion protein associated with virulence [54], a thermonuclease, DNA methylase, DEAD-box helicase, and a type III restriction enzyme and modification system. Moreover, plasmids pLMG2-8 to 10 and pLM80 all contained an additional region encoding the multidrug resistance proteins EbrA and EbrB. This region was also identified in pLMG2-3 from G2 Sub2 and in two G1 plasmids (pLMG1-10 and 11).

### 2.5. Expression of the L. monocytogenes Plasmid-Encoded Genes During Growth Under Salt and Acid Stress Conditions

To investigate the hypothesis that plasmids contribute to enhanced salt and acid tolerance in *L. monocytogenes*, the expression levels of a number of plasmid-encoded genes were investigated in three *L. monocytogenes* strains during growth in BHIB with 6% NaCl or adjusted to pH 5. The investigated genes were selected based on being prevalent in both G1 and G2 plasmids or being uniquely present in a high number of plasmid types from a single plasmid group. Furthermore, genes were chosen based on having functions with probable roles in bacterial stress response.

**Figure 5:**
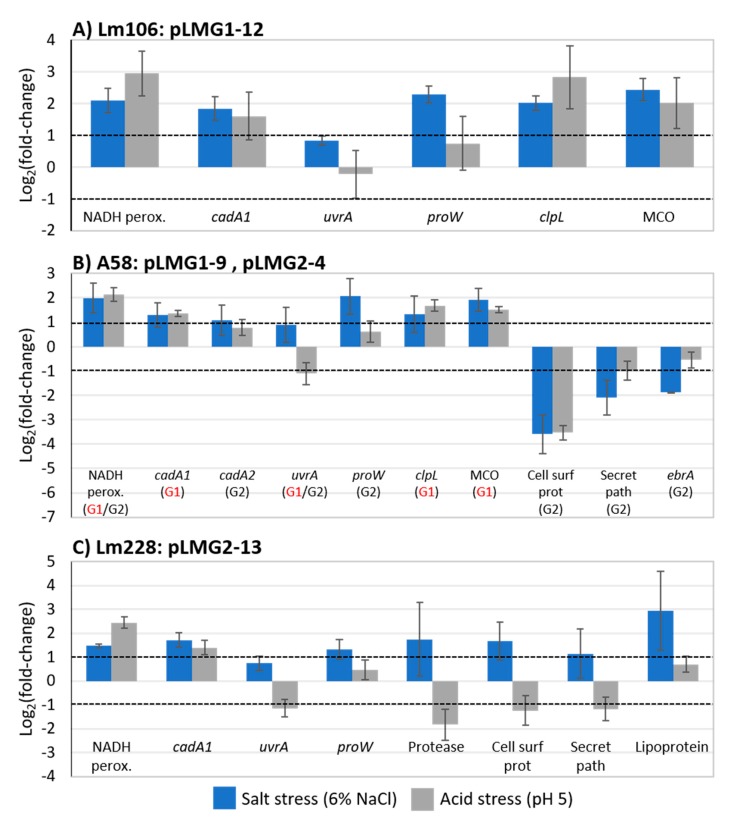
The differential expression of *L. monocytogenes* plasmid-encoded genes in three different strains ((**A**)—Lm106; (**B**)—A58; (**C**)—Lm228) subjected to salt (BHIB+6% NaCl, 30 °C) and acid (BHIB pH 5, 30 °C) stress in comparison to the control (BHIB, 30 °C). RNA was extracted from mid-exponential phase cells. Bar heights represent average log_2_ fold changes and error bars denote standard deviations (*n =* 4). Bars with >2-fold increase (>1 log_2_) or decrease (< −1 log_2_) in expression were considered significantly different (*p* < 0.05) as shown by the black-dashed line. BHIB: brain heart infusion broth; MCO: multicopper oxidase; Cd: cadmium; Cell surf prot: cell surface protein; Secret path: secretion pathway E.

In strain Lm106, the plasmid-encoded (pLMG1-12) genes for NADH peroxidase, a cadmium-transporting ATPase, ProW, ClpL, and multicopper oxidase (MCO) were all upregulated >2-fold during growth in 6% NaCl (Figure 5A). Similar results were seen for growth in pH 5 media with the exception of *proW* which had a <2-fold level of induction (Figure 5A). *uvrA* encoding excinuclease ABC subunit A, was the only gene not upregulated >2-fold in Lm106 under either stress condition. Near identical gene expression trends were seen for the same genes on two plasmids (pLMG1-9 and/or pLMG2-8) found in strain A58 (Figure 5B). A58 additionally contained two cadmium ATPases (one on each plasmid, *cadA1* and *cadA2*), and while both were upregulated during growth in 6% NaCl and at pH 5, the pLMG2-8 version (*cadA2*) was induced to a lesser extent compared to the version (*cadA1*) found in pLMG1-9. The presence of *cadA1* and *cadA2* on separate plasmids instead of on the same plasmid was also mentioned in a recent review [25]. However, at this time it is not known whether one *cadA* gene confers better protection against cadmium and/or stress than the other. Three additional G2-specific plasmid-encoded genes (cell surface protein, secretion pathway E, and *ebrA*) were downregulated in strain A58 under both stress conditions (Figure 5B).

Like in Lm106 and A58, genes encoding NADH peroxidase and cadmium ATPase were again upregulated >2-fold in Lm228 under both stress conditions and *proW* was upregulated >2-fold in response to only salt stress (Figure 5C). A gene encoding a lipoprotein was also significantly upregulated under salt but not acid stress conditions (Figure 5C). The lipoprotein shares 99% of the protein identity to multiple *L. monocytogenes* cell wall hydrolases that aid in the growth and development of cell walls and can be major determinants of growth rate and cell wall architecture [55]. *uvrA* again was not significantly upregulated under salt stress conditions and was downregulated in Lm228 under acid stress conditions (Figure 5C). This gene was originally selected for its known roles in DNA repair, specifically, DNA damage caused by UV radiation, oxidative stress, and bile salts [56,57,58]. Genes encoding a protease, cell surface protein, and secretion pathway E were all significantly upregulated in Lm228 under salt stress conditions, but significantly downregulated under acid stress conditions (Figure 5C). It should be noted that different versions of the cell surface and secretion pathway E proteins existed in strains A58 and Lm228 which may explain why differences in expression were observed. Cell surface proteins have been shown to participate in a broad range of activities including environmental signaling, surface and cell adhesion, and pathogenesis [59,60]. The protease shared 99% protein identity with an intramembrane metalloprotease which serves to maintain homeostasis of cell surface components by cleaving damaged or misfolded proteins that can occur following stress [61]. Secretion pathway protein E, on the other hand, is typically involved in triggering a host response that promotes virulence in *L. monocytogenes* [62]. Its putative role in salt tolerance remains to be discovered.

Plasmid-encoded NADH peroxidases were activated in all three *L. monocytogenes* strains during growth under salt and acid stress conditions. NADH peroxidases have been proven to mitigate oxidative stress by decomposing hydrogen peroxide which accumulates in cells during aerobic growth [63,64]. Our results, therefore, suggest that the cells were experiencing enhanced levels of oxidative stress under both salt and acid stress conditions and that the presence of plasmid-encoded NADH peroxidases may be beneficial for surviving such stresses.

*proW* encodes an L-proline glycine betaine ABC transport system permease protein. Both L-proline and glycine betaine are compatible solutes that have been shown to accumulate in multiple bacteria and plants during osmotic stress exposure, usually through membrane transportation as opposed to de novo synthesis [65,66,67,68,69,70]. These solutes are believed to help stabilize proteins against denaturation as well as counteract the outflow of water from cells under hypertonic growth conditions [71]. It is therefore not surprising that plasmid-encoded *proW* was upregulated in all three *L. monocytogenes* strains under salt stress conditions. The uptake of compatible solutes has not previously been associated with acid stress and our findings also support this. On the contrary, Clp ATPases have been shown to be associated with bacterial acid, bile salt, and heat stress responses as well as virulence [72,73,74,75,76]. These enzymes are widely conserved bacterial chaperone proteins which have critical roles in refolding and degrading damaged cell proteins [73]. Accordingly, Clp ATPase was significantly upregulated under both stress conditions in Lm106 and A58 in which plasmid-encoded versions of the gene were present. Similarly, in another study, plasmid-encoded Clp ATPases have been shown to enhance the heat resistance of *L. monocytogenes* strains [76].

A plasmid-encoded multicopper oxidase was also upregulated in *L. monocytogenes* during growth under salt and acid stress conditions. Aside from the proven role of these enzymes in copper homeostasis, they also show enhanced oxidase activity for a wide range of substrates and participate in transmembrane iron transport [77,78]. Since iron homeostasis and responses to oxidative stress are coordinated [79], multicopper oxidase may have a role in mediating the additional oxidative stress imposed by salt and acid stress conditions. Like many of the genes found in *L. monocytogenes* plasmids, multicopper oxidases have not been found in *L. monocytogenes* chromosomes but are made available to this pathogen through plasmid harbourage.

The roles of cadmium ATPases in cadmium detoxification and resistance have been thoroughly studied in several bacteria [13,22,80], but their putative functions in bacterial salt and acid tolerance remain unknown. Casey et al. [81] found that a plasmid-encoded cadmium ATPase was upregulated 2.45-fold in *L. monocytogenes* cells exposed to 4 ppm of benzethonium chloride, a quaternary ammonium-based sanitizer. It is, therefore, possible that like multicopper oxidase, cadmium ATPases have the ability to act on additional substrates. However, unlike multicopper oxidase, cadmium ATPases are also found within transposons embedded in *L. monocytogenes* chromosomes [36].

Multidrug efflux pumps, such as EbrAB investigated in this study, have been shown to have roles in bacterial virulence, as well as bile and sanitizer resistance [11,82,83]. However, despite bile containing high levels of salt, we did not observe the induction of this gene during the growth of *L. monocytogenes* in 6% salt or pH 5 media (Figure 5B).

### 2.6. Stress Tolerance Comparisons of Wildtype and Plasmid-Cured Strains

To further investigate if plasmid-encoded genes were responsible for the significantly enhanced acid tolerance of plasmid-harbouring strains and the enhanced salt tolerance of G2 plasmid strains observed in our previous study [30], the growth rates of two plasmid-cured strains (Lm10_PC, Lm20_PC) were compared to those of their wildtype strains (Lm10, Lm20) under the control and salt and acid stress conditions.

**Figure 6:**
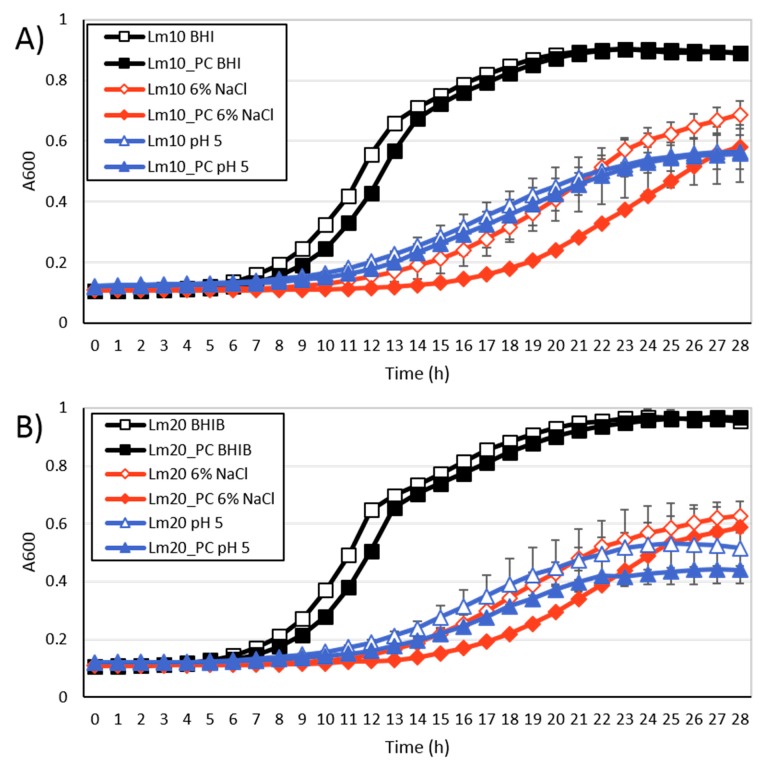
The growth of wildtype *L. monocytogenes* strains and their plasmid-cured (PC) counterparts in brain heart infusion broth (BHIB) +6% NaCl, and BHIB pH 5 at 25 °C. (**A**) Lm10 and Lm10_PC, (**B**) Lm20 and Lm20_PC. Data points denote the averages of the three replicates and error bars represent standard deviations.

When grown in BHIB at 25 °C, both Lm10 and Lm20 exhibited maximum growth rates and cell densities that were similar (*p* > 0.05) to those of the plasmid-cured strains (Figure 6, Table A1). However, as seen in Figure 6, the plasmid-cured strains exhibited longer lag phase durations (~1 h longer, *p* ≤ 0.001) compared to the wildtype strains. This is the opposite of what was hypothesized, as plasmid harbourage is often thought, and has been shown by some studies, to pose an additional burden on cells during replication [29,84,85]. However, Logue et al. [86] also reported that a plasmid-cured strain of *Yersinia enterocolitica* had a longer lag-phase duration than its wildtype strain when grown in BHIB at 25 °C, but that the exponential growth rates were comparable. This may suggest that plasmid-encoded genes also have active roles in normal cell growth, in particular, the processes involved in transitioning from the lag phase to exponential growth.

When the wildtype and plasmid-cured strains were grown in 6% NaCl and pH 5 media, the results were similar to those observed for growth in BHIB. In 6% NaCl, the plasmid-cured strains had lag phase durations that were significantly longer (3–4 h, *p* ≤ 0.012) than those obtained for the wildtype strains (Figure 6, Table A1). In pH 5 media, the lag phase duration of Lm20_PC was significantly (*p* = 0.050) longer by 2 h compared to Lm20, while no significant difference was found between that for Lm10_PC and Lm10 (Figure 6, Table A1). Again, the maximum growth rates and maximum cell densities did not differ (*p* > 0.05) between plasmid-cured and wildtype strains in 6% NaCl or pH 5 media. Based on these findings, it appears that plasmid harbourage may be more beneficial for growth in 6% NaCl than at pH 5. However, since the plasmid-cured strains exhibited longer growth phases in BHIB as well, it is difficult to conclude that this difference was not more emphasized under salt stress conditions.

Naditz et al. [18] recently compared the survival of three sets of wildtype and plasmid-cured *L. monocytogenes* strains under oxidative (0.01% H_2_O_2_), heat (55 °C), salt (15% NaCl), and acid (pH 3.4) stress conditions and found that the wildtype strains exhibited significantly enhanced survival in all cases. Here, the benefit of plasmid harborage was more evident than in the present study, likely as a result of the different strains and conditions used. In the present study, less severe levels of salt and acid stress were employed in order to further explore the findings of our previous study and to gain information pertaining to how plasmids may influence the growth ability of *L. monocytogenes* in ready-to-eat foods. However, based on both our findings and those of Naditz et al. [18], the benefits of plasmid-harborage with regards to food-related stresses, may only be noticeable under more lethal stress conditions.

## 3. Conclusions

This study examined both the genetic similarities and differences between two *L. monocytogenes repA* sequence-based plasmid groups (G1 and G2) and the putative roles of specific plasmid-encoded genes in the tolerance of *L. monocytogenes* to salt and acid stress. To the best of our knowledge, this is the largest comparison of *L. monocytogenes* plasmids and their associated strains, which has been conducted to date. Our results showed that G1 and G2 plasmids contained many similar but also unique sets of genes. G1 plasmids were significantly smaller than G2 plasmids but contained a larger diversity of genes across plasmid types. With that said, G1 plasmids formed one main phylogenic cluster while G2 plasmids formed two distinct subgroups with subgroup 2 plasmids containing multidrug resistance and numerous virulence-associated genes. Across all *L. monocytogenes* plasmids was an abundance of genes with putative roles in bacterial stress responses. qPCR revealed that a number of these genes are activated in *L. monocytogenes* during growth under salt and acid stress conditions. Specifically, an NADH peroxidase, cadmium ATPase, multicopper oxidase, and ClpL chaperone protein were all upregulated (>2-fold) under both conditions and an osmolyte transporter (*ProW*) was upregulated under salt conditions while a G2-specific lipoprotein was upregulated under acid stress conditions. A comparison of the growth rates of two plasmid-cured strains to their wildtype strains during growth under both stresses did not reveal any differences in growth rates between the strains but demonstrated longer lag phases for the plasmid-cured strains under all conditions tested.

Collectively, this work suggests that plasmids may have roles in facilitating the growth and/or survival of *L. monocytogenes* under salt and acid stress conditions. This would pose an additional concern for the food industry where food-related *L. monocytogenes* isolates have already been shown to have higher rates of plasmid harbourage. Furthermore, plasmid harbourage may also be associated with improved gastrointestinal survival as salt and acid stress conditions are frequently encountered by *L. monocytogenes* along this route. Similarly, the possible roles of the many plasmid-encoded virulence-associated and hypothetical proteins also warrant further investigation.

Lastly, 26 strains from Canada and Switzerland and covering many different serotypes, harboured identical plasmids highlighting the astounding conservation of *L. monocytogenes* plasmids globally. It is reasonable to expect that plasmids, which support the growth and/or survival of *L. monocytogenes,* will continue to spread in the population. As whole-genome sequencing is becoming increasingly more affordable and popular, an emphasis should be placed on screening *L. monocytogenes* sequences for plasmids and making global comparisons to determine their spread. This will allow us to not only be able to track the evolution of *L. monocytogenes* plasmids more easily, but also monitor the overall prevalence of plasmid-harbourage among food-related isolates.

## 4. Materials and Methods

### 4.1. Strains and Culture Conditions

A total of seven *L. monocytogenes* strains were used in this study (Table 5). Five strains were previously sequenced and characterized for food-related stress tolerances [30] and two were plasmid-cured versions of two of these five strains. Plasmid-cured strains were previously obtained [87] using a protocol adapted from Lebrun et al. [7]. In short, the strains were repeatedly passaged (up to 6×) on brain heart infusion (BHI) agar (Difco, Fisher Scientific, Ottawa, Ontario, Canada) under an elevated temperature of 45 °C, and three colonies from each passage were tested for plasmid-loss using PCR-based assays. The strains were stored at −80 °C in BHI broth (BHIB; Difco, Fisher Scientific, Ottawa, Ontario, Canada) with 20% glycerol and routinely cultured at 30 °C on BHI agar plates.

### 4.2. Genetic Comparisons of L. Monocytogenes Plasmids

A total of 93 previously concatenated plasmid sequences originating from 92 *L. monocytogenes* strains [30], were analyzed in this study. In short, all contigs of *repA*-positive strains were aligned to the closed genome of Lm EDG-e using *Progressive Mauve* in Mauve v2.4.0 [88]. Contigs not aligning to EGD-e were excluded as plasmid-associated if they contained chromosomal DNA elements (ex. rRNA, tRNA). The remaining putative plasmid contigs were then put through Blastn on the NCBI server (https://www.ncbi.nlm.nih.gov) to determine if they shared any similarity with other published plasmid sequences. In all cases this was true and the resulting plasmid-contigs were extracted from the other whole-genome sequence contigs for further analysis. Blastn was also used to group together strains from our previously published sequence collection on NCBI (BioProject PRJNA329415), that contained similar or identical plasmid contigs. Plasmid sequences sharing >99% nucleotide identity and differing by at most one mobile element gene, were categorized as belonging to a single plasmid type. These sequences were also previously [30] determined to belong to one of two plasmid groups (G1 and G2), based on the results of a *repA* phylogeny that was constructed as described in Kuenne et al. [16]. *repA* was used for the basis of the phylogeny as it encodes a protein that controls plasmid copy-numbers in bacteria and is one of the few genes found in all plasmids.

One representative sequence of each plasmid type was annotated using ClassicRAST on The RAST v2.0 server [89,90,91] with the taxonomy ID 1639 for *L. monocytogenes*. The types of genes found in the different plasmid types and within plasmid groups (G1 and G2) were then compared. Plasmid types were named according to their *repA* group and the size of the plasmid compared to others in our sequence collection.

FigTree v1.4.3 (http://tree.bio.ed.ac.uk/software/figtree/) was used to visualize a Neighbour Joining phylogenetic guide tree of the various plasmid types based on a sequence alignment produced using *Progressive Mauve* in Mauve v2.4.0 [88]. For comparison, eight additional plasmid sequences (pLM80, pLM1-2bUG1, pLM5578, pCT100, pLM7UG1, pLm33, pLGUG1, pLI100) used in Kuenne et al. [16] were also included in the alignment and tree. Noteworthy, pLI100 is from an *L. innocua* strain and pLGUG1 is from an *L. grayi* strain while all others are from *L. monocytogenes* strains of human or food origins.

Lastly, plasmid types were screened for antimicrobial resistance genes using ResFinder 2.1 available on the Center for Genomic Epidemiology, Technical University of Denmark server (https://cge.cbs.dtu.dk/services/ResFinder-2.1/), using 70% gene identity and 60% coverage as cutoffs.

### 4.3. RNA Isolation and Real-Time qPCR Analysis

Three *L. monocytogenes* strains (A58, Lm106, Lm228) were used to evaluate the expression levels of plasmid-encoded genes under salt and acid stress conditions. Lm106 and Lm228 were selected to represent G1 and G2 plasmid strains, respectively, as both of their plasmids contained a large number of genes (21–22 with functions) that were highly prevalent (>50%) among plasmids belonging to these groups. Strain A58 was included because it uniquely contained both a G1 and a G2 plasmid and we were interested in comparing the expression levels of similar genes found in both plasmids.

One colony from each of the three *L. monocytogenes* strains was inoculated into 10 mL of BHIB and incubated at 30 °C for 18 h. The cultures were then diluted to 10^7^ CFU/mL in either fresh BHIB, BHIB+6% NaCl (w/w), or BHIB adjusted to pH 5 and again incubated at 30 °C. At the mid-exponential phase (10^8^ CFU/mL, A_600nm_ = 0.08–0.1), the cellular metabolism was halted by adding 10% phenol:chloroform (Fisher Scientific) in ethanol solution pre-chilled to −80 °C in a 1:10 volume to the sample. The tubes were vortexed briefly and then centrifuged immediately for 10 min at 4696× *g* and 0 °C. The supernatants were removed and the resulting pellets were stored at −80 °C. Four biological replicates were performed for each strain and treatment combination.

The total RNA was isolated and purified using the PowerMicrobiome™ RNA Isolation kit (MO BIO Laboratories, Carlsbad, CA, USA) per the manufacturer’s protocol. RNA integrity numbers (RINs) were determined using the 2100 Bioanalyzer (Agilent, Santa Clara, CA, USA). Samples with a RIN between 9.7 and 10 were converted to cDNA using the QuantiTect Reverse Transcription Kit (Qiagen, Valencia, CA, USA) per the manufacturer’s protocol. Primers for select genes (Table A2) were designed using Primer3 Plus available on the NCBI server (https://www.ncbi.nlm.nih.gov/tools/primer-blast/) and our draft whole-genome sequences for the strains. qPCR was conducted in a CFX96 Touch^TM^ Real-Time PCR Detection System (BioRad, Hercules, CA, USA) using SsoAdvanced^TM^ Universal SYBR Green ^®^ Supermix (BioRad, Hercules, CA, USA). The thermocycling parameters used were as follows: initial denaturation for 30 s at 98 °C, followed by 40 cycles of denaturation for 30 s at 95 °C and elongation for 40 s at 57 °C for 40 s. Melting curves were subsequently performed at 65 °C using 0.5 °C increments. The relative expression levels of the plasmid-encoded genes were calculated using the 2^−ΔΔCT^ method [92] with 16S rRNA as the reference gene [93]. Genes with an average fold change >1 or < −1 log_2_ were considered statistically induced or suppressed, respectively.

### 4.4. Stress Tolerance Comparisons of Wildtype and Plasmid-Cured Strains

Wildtype (Lm10, Lm20) and plasmid-cured strains (Lm10_PC, Lm20_PC) were compared on their ability to grow under salt and acid stress conditions using previously described protocols [30]. Cultures were grown to stationary phase in BHIB at 30 °C and then diluted in either BHIB+6% (*w*/*w*) NaCl or BHIB was adjusted to pH 5 to achieve a final concentration of 10^7^ CFU/mL in 96-well plates (Costar™ clear polystyrene, Fisher Scientific) that were then incubated at 25 °C in a microplate reader with the absorbance set to 600 nm (Spectramax, V6.3; Molecular Devices, Sunnyvale, CA, USA). The resulting growth curves were fitted to the Baranyi and Roberts model [94] using DMfit (v3.5) available on the ComBase browser (http://browser.combase.cc/DMFit.aspx). The entire experiment was repeated three times and model parameters of the three biological replicates were compared between the wildtype and plasmid-cured (PC) strains using Student’s *t*-test with a 95% confidence level (*p* < 0.05).

## Figures and Tables

**Table 1:** The characteristics of group 1 (G1) *Listeria* plasmids analyzed in this study and their associated strains. See Table S1 for plasmid contig Genbank accession numbers.

Plasmid Type	Sub-Group ^a^	# of Strains ^b^	Strain Origin(s) ^c^	Serotype ^d,e^ or Species	Clonal Complex ^d^	Plasmid Size (bp) ^d,f^	# of Plasmid Contigs	Plasmid %GC	# of Predicted Genes	# of MGEs ^g^
pLM33 ^ǂ^	MC	-	-	1/2b	-	32307	closed	36.16	36	9
pLMG1-1	MC	3	CH	1/2a, 1/2c (2)	9	25605	1	36.85	29	8
pLMG1-2	MC	1	CH	1/2c	9	38056	3	36.85	42	8
pLMG1-3	MC	1	AB	1/2a	Singleton (ST 839)	40730	3	36.85	48	6
pLMG1-4	MC	3	AB	1/2c	9	44350	3	36.85	54	11
pLMG1-5	MC	7	AB	1/2c	9	48409 (6), 48460	3	36.85	52	12
pLMG1-6	Outlier	4	BC	4b	6	54735, 54736 (3)	5	34.77	64	9
pCT100 ^ǂ^	Outlier	-	-	*L. innocua*	-	37279	closed	34.07	34	6
pLM1-2bUG1 ^ǂ^	MC	-	-	1/2b	-	57780	closed	36.04	63	16
pLMG1-7	MC	26	AB (18), BC (4), CH (4)	1/2a (17), 1/2b (6), 1/2c (2), 3b	3 (5), 5 (2), 7 (9), 9 (3), 11 (5), 89, 193	57082, 57083 (25)	3	36.04	64	13
pLM7UG1 ^ǂ^	MC	-	-	7	-	50100	closed	35.48	55	13
pLMG1-8	MC	3	AB, BC (2)	1/2c	9	58105	3	36.65	69	13
pLMG1-9*	MC	1	AB	1/2b	5	62258	2	35.58	75	17
pLMG1-10	MC	1	AB	1/2a/3a	199	70385	3	36.36	83	17
pLMG1-11	G2 Sub2	1	BC	1/2c	9	75351	7	36.85	88	13
pLMG1-12	MC	2	AB, BC	1/2b	5	78240, 78245	4	36.14	95	18
pLMG1-13	Outlier	5	BC	1/2a	11	87487 (2), 87488, 87574 (2)	1	34.38	113	15

^ǂ^ Plasmids highlighted in yellow were retrieved from Genbank and are included in this study for comparison purposes. * indicates one of two plasmids found in a single strain. ^a^ MC refers to the main G1 cluster shown in Figure 1. ^b^ Number of strains from our collection that contained a particular plasmid type. ^c^ AB = Alberta, Canada; BC = British Columbia, Canada; CH = Switzerland. ^d^ Numbers in brackets denote the number of plasmids belonging to each characteristic when more than one is listed in a cell. ^e^ Serotypes refer to *L. monocytogenes* strains only. ^f^ Plasmid size refers to the combined size of the assembled contigs contributing to each plasmid. ^g^ Mobile genetic elements (MGEs) included genes predicted to encode a mobile element protein, recombinase, transposase, integrase, invertase, or resolvase.

**Table 2:** The characteristics of group 2 (G2) *Listeria* plasmids analyzed in this study and their associated strains. See Table S1 for plasmid contig Genbank accession numbers. Plasmids highlighted in yellow were retrieved from Genbank and are included in this study for comparison purposes.

Plasmid Type	Sub-Group ^a^	# of Strains ^b^	Strain Origin(s) ^c,d^	Serotype ^d,e^ or Species	Clonal Complex ^d^	Plasmid Size (bp) ^d,f^	# of Plasmid Contigs	Plasmid %GC	# of Predicted Genes	# of MGEs ^g^
pLMG2-1	Sub1	1	AB	1/2a	8	55472	1	37.55	63	1
pLMG2-2	Sub1	1	CH	1/2a	121	61053	1	36.85	64	4
pLM80 ^ǂ^	Sub 2	-	-	4b	-	81588	2	37.54	88	11
pLMG2-3	Sub1	6	BC	1/2a (4), 3a (2)	321	66447	3	36.85	74	6
pLMG2-4	Sub2	6	AB	1/2a (6)	8	77109, 77221 (2), 77229 (3)	1	36.85	83	9
pLM5578 ^ǂ^	Sub 1	-	-	1/2a	-	77054	closed	36.59	76	11
pLMG2-5	Sub1	2	AB	1/2a	8, singleton (ST 1018)	77249	1	36.85	83	10
pLGUG1 ^ǂ^	Sub 2	-	-	*L. grayi*	-	79249	closed	36.78	99	8
pLMG2-6	Sub1	2	AB	1/2c	9	81510	2	36.85	87	5
pLMG2-7	Sub2	3	AB, BC (2)	1/2a, 1/2b (2)	7, 88 (2)	81644	1	36.85	92	7
pLMG2-8*	Sub2	1	AB	1/2b	5	87369	3	37.71	97	10
pLMG2-9	Sub2	1	BC	1/2a	155	89025	2	37.55	99	10
pLMG2-10	Sub2	4	AB, BC, CH (2)	1/2a (2), 1/2b (2)	5 (2), 204 (2)	90543	3	36.85	99	11
pLI100 ^ǂ^	Sub 2	-	-	*L. innocua*	-	81905	closed	35.52	84	24
pLMG2-11	Sub1	6	AB (5), BC	1/2a	8	92204 (5), 99205	1	36.85	98	10
pLMG2-12	Sub1	1	AB	1/2a	8	98358	2	36.85	108	12
pLMG2-13	Sub1	1	CH	1/2b	59	107184	2	36.85	120	11

^ǂ^ Plasmids highlighted in yellow were retrieved from Genbank and are included in this study for comparison purposes. * indicates one of two plasmids found in a single strain. ^a^ Sub 1 and Sub 2 refer to the two G2 clusters shown in Figure 1. ^b^ Number of strains from our collection that contained a particular plasmid type. ^c^ AB = Alberta, Canada; BC = British Columbia, Canada; CH = Switzerland. ^d^ Numbers in brackets denote the number of plasmids belonging to each characteristic when more than one is listed in a cell. ^e^ Serotypes refer to *L. monocytogenes* strains only. ^f^ Plasmid size refers to the combined size of the assembled contigs contributing to each plasmid. ^g^ Mobile genetic elements (MGEs) included genes predicted to encode a mobile element protein, recombinase, transposase, integrase, invertase, or resolvase.

**Table 3:** The predicted proteins uniquely observed in group 1 plasmids. Plasmid types highlighted in blue did not belong to the main G1 cluster in Figure 1. Yellow cells indicate the presence of one of the predicted proteins on a plasmid type while orange cells indicate the presence of two of the same protein on the plasmid.

Predicted Protein	G1 Plasmid Types												
	1	2	3	4	5	6	7	8	9	10	11	12	13
Acyltransferase family protein													
Alcohol dehydrogenase													
Arsenic efflux pump protein													
ATPase involved in DNA repair													
Cadmium resistance protein													
Copper-transporting ATPase													
CRISPR-associated protein MTH1087													
Dihydroxyacetone kinase, ATP-dependent													
DUF1706 domain-containing protein													
Epsilon antitoxin													
Glutathione-dependent formaldehyde dehydrogenase													
Glycerol dehydrogenase													
Glycerol kinase													
Hypothetical lambda repressor-like, DNA-binding													
Integral membrane protein													
Lmo0466 protein													
Lmo2276 protein													
Membrane proteins related to metalloendopeptidases													
Mercuric ion reductase													
Mercuric resistance operon regulatory protein													
Methyl-accepting chemotaxis protein													
Multi antimicrobial extrusion (MATE) family transporter													
Myosin heavy chain, nonmuscle type B													
Na(+)/H(+) antiporter													
Organomercurial lyase													
Oxidoreductase (putative)													
Permease of the drug/metabolite transporter superfamily													
Phage protein													
Phosphate regulon transcriptional regulatory protein PhoB													
Phosphoenolpyruvate-dihydroxyacetone phosphotransferase ADP-binding subunit DhaL													
Phosphoenolpyruvate-dihydroxyacetone phosphotransferase dihydroxyacetone binding subunit DhaK													
Phosphoenolpyruvate-dihydroxyacetone phosphotransferase subunit DhaM													
Predicted transcriptional regulator of pyridoxine metabolism													
Prophage LambdaSa2, site-specific recombinase													
Protein involved in cell division													
Protoporphyrinogen IX oxidase, novel form, HemJ													
pXO2-10													
RelB/StbD replicon stabilization protein (antitoxin to RelE/StbE)													
RelE/StbE replicon stabilization toxin													
RepB													
Replication-associated protein RepB													
Rlx-like protein													
Site-specific recombinase, DNA invertase													
Site-specific recombinase, phage integrase family													
Sortase A, LPXTG specific													
Tn916, transcriptional regulator, putative													
Transcriptional regulator, PadR family													
Transcriptional regulator, XRE family													
Transcriptional repressor, BlaI/MecI family													
Transposase, IS204/IS1001/IS1096/IS1165													
Type I restriction-modification system, restriction subunit R													
Zeta toxin													

**Table 4:** The predicted proteins uniquely observed in group 2 plasmids. Plasmid types highlighted in blue belong to the G2 Sub 1, all others belong to the G2 Sub 2. Yellow cells indicate the presence of one of the predicted proteins on a plasmid type.

Predicted Protein	G2 Plasmid Types												
	1	2	3	4	5	6	7	8	9	10	11	12	13
ABC transporter													
Cell surface protein													
Chromosome (plasmid) partitioning protein ParA													
Conjugation protein, TraG/TraD family, (pXO2-16)													
Conserved hypothetical protein													
General secretion pathway protein E													
Hypothetical protein, (pXO1-65)													
Hypothetical protein, (pXO2-28)													
Invasion associated protein p60													
Lipoprotein, NLP/P60 family													
Membrane protein, putative, (pXO2-14)													
Membrane-bound protease, CAAX family													
Phage protein lin1266													
Pli0009 protein													
Pli0068 protein													
Secreted antigen GbpB/SagA/PcsB, putative peptidoglycan hydrolase													
Thermonuclease													
Tn5252, Orf 21 protein, internal deletion													
TolA protein													
TraG/TraD family protein													
Type IV secretory pathway, VirD4 components													
Type V secretory pathway, adhesin AidA													

**Table 5:** The *L. monocytogenes* strains used in this study.

Strain	Origin	Serotype	Plasmid Type ^a^	Notable Characteristics
A58	AB	1/2b	pLMG1-9 pLMG2-4	- Acid tolerant - Contains two plasmids
Lm10	BC	1/2a	pLMG2-9	
Lm10_PC		1/2a	N/A	- Plasmid-cured Lm10
Lm20	BC	1/2c	pLMG1-11	
Lm20_PC		1/2c	N/A	- Plasmid-cured Lm20
Lm106	BC	1/2b	pLMG1-12	
Lm228	CH	1/2b	pLMG2-13	- Acid/salt tolerant - Harbours the largest plasmid in our strain collection

^a^ G1 and G2 within the plasmid types denote the group that the plasmid belongs to as determined by a *repA* phylogeny. Origin: AB = Alberta, Canada; BC = British Columbia, Canada; CH = Switzerland.

## Supplementary Materials

The following are available online at https://www.mdpi.com/2072-6651/11/7/426/s1. Table S1: Complete list of plasmid-positive strains in our *L. monocytogenes* collection and their associated characteristics and plasmid contig Genbank accession numbers, Table S2: Summary of all predicted genes identified on each *L. monocytogenes* plasmid type.

## Appendix A

**Table A1:** Model parameters of the growth kinetics of wildtype *L. monocytogenes* strains and their plasmid-cured (PC) counterparts in brain heart infusion broth (BHIB) +6% NaCl, and BHIB pH 5 at 25 °C.

Condition	Strain	LPD (h)	µ_max_ (A_600nm_/h)	N_max_ (A_600nm_)
BHIB (25 °C) Control	Lm10	7.38 ± 0.08 ^a^	0.09 ± 0.00 ^a^	0.89 ± 0.01 ^a^
	Lm10_PC	8.46 ± 0.05^b^	0.09 ± 0.00 ^a^	0.88 ± 0.01 ^a^
	Lm20	6.72 ± 0.05 ^a^	0.09 ± 0.00 ^a^	0.94 ± 0.02 ^a^
	Lm20_PC	7.59 ± 0.17 ^b^	0.09 ± 0.00 ^a^	0.94 ± 0.02 ^a^
BHIB + 6% NaCl	Lm10	13.39 ± 1.50 ^a^	0.05 ± 0.00 ^a^	0.70 ± 0.06 ^a^
	Lm10_PC	17.17 ± 0.10 ^b^	0.05 ± 0.00 ^a^	0.60 ± 0.01 ^a^
	Lm20	12.75 ± 0.64 ^a^	0.04 ± 0.00 ^a^	0.62 ± 0.05 ^a^
	Lm20_PC	15.79 ± 0.38 ^b^	0.04 ± 0.00 ^a^	0.60 ± 0.02 ^a^
BHIB pH 5	Lm10	10.13 ± 0.28 ^a^	0.03 ± 0.01 ^a^	0.56 ± 0.05 ^a^
	Lm10_PC	11.17 ± 0.78 ^a^	0.03 ± 0.01 ^a^	0.56 ± 0.09 ^a^
	Lm20	9.70 ± 1.22 ^a^	0.04 ± 0.02 ^a^	0.71 ± 0.32 ^a^
	Lm20_PC	12.01 ± 0.76 ^b^	0.04 ± 0.01 ^a^	0.55 ± 0.19 ^a^

LPD = lag phase duration. µmax = maximum growth rate. Nmax = maximum absorbance reached. Values represent mean ± standard deviation (*n =* 3). Values in the same column followed by different superscript letters are significantly different between pairs of wildtype and plasmid-cured strains (i.e., Lm10 vs. Lm10_PC).

**Table A2:** Primers used for quantitative PCR of plasmid-encoded genes.

Target Gene	Primer Sequence	Amplicon Length (bp)	Source
16S rRNA	Fw- TTAGCTAGTTGGTAGGGT Rv- AATCCGGACAACGCTTGC	550	[93]
*ProW*	Fw- GGAAGCCGCATTGGATGTTC Rv- CCTCGTAGCCACCAAAGACA	78	This study
NADH peroxidase	Fw- ACTGGCGAAGGTGTGAAAGA Rv- GGACGAACCCCAACACTGAT	116	This study
*clpL*	Fw- TGGGCACTTAACGGATGGAC Rv- TGATCTCGTTGCTCGTCACC	117	This study
Cell surface protein (A58 G2 plasmid)	Fw- GGTGGAACTAACCGATGCGA Rv- TGACTTGCTTGTTCGTTGCG	71	This study
Cell surface protein (Lm228)	Fw- CTATCGCGTGACCAAACGTG Rv- GGCGCAGCTTTCATTTCACT	71	This study
Lipoprotein	Fw- CTGCTTGCGGCGATTTCTTT Rv- TGCCGACATCATTCCACCAA	90	This study
Secretion system E (A58 G2 plasmid)	Fw- GCCCCTAAGCCGATAGAACG Rv- ACCCGTTCCAGTTTCTCCAG	84	This study
Secretion system E (Lm228)	Fw- TCTGTTGGGGCAAAAGCATC Rv- TGCAACAATGGCTTTGCGTT	77	This study
Multicopper oxidase	Fw- ACAGTCCTTGTGAATGGGAAAGT Rv- AAATCCCGAGCATTGGAGCC	95	This study
Cadmium ATPase (*cadA1*, Lm106 and Lm228)	Fw- AAACTGGGAAACGTGGGTGT Rv- CATTTCCAATGGCGGTGACG	107	This study
Cadmium ATPase (*cadA2*, A58 G2 plasmid)	Fw- AGAACAAGGGAAAACCGCCA Rv- TACTTCATCCGCGACAGCAA	75	This study
*uvrA*	Fw- AGGCTGTATGTCACGGTTGG Rv- GCCACTCCGCAATACCTGAT	100	This study
*ebrA*	Fw- GGTATGGGGAGTGCTTTTTACCT Rv- TCCTCCACCAGACCATATTGC	87	This study
Membrane-bound protease	Fw- CGGTCATTCCCCTAATCAACCT Rv- TTGCCATACAAACCCATGTGC	73	This study
